# A Causal Inference Model Explains Perception of the McGurk Effect and Other Incongruent Audiovisual Speech

**DOI:** 10.1371/journal.pcbi.1005229

**Published:** 2017-02-16

**Authors:** John F. Magnotti, Michael S. Beauchamp

**Affiliations:** Department of Neurosurgery and Core for Advanced MRI, Baylor College of Medicine, Houston, Texas, United States of America; Harvard University, UNITED STATES

## Abstract

Audiovisual speech integration combines information from auditory speech (talker’s voice) and visual speech (talker’s mouth movements) to improve perceptual accuracy. However, if the auditory and visual speech emanate from different talkers, integration decreases accuracy. Therefore, a key step in audiovisual speech perception is deciding whether auditory and visual speech have the same source, a process known as causal inference. A well-known illusion, the McGurk Effect, consists of incongruent audiovisual syllables, such as auditory “ba” + visual “ga” (AbaVga), that are integrated to produce a fused percept (“da”). This illusion raises two fundamental questions: first, given the incongruence between the auditory and visual syllables in the McGurk stimulus, why are they integrated; and second, why does the McGurk effect not occur for other, very similar syllables (*e*.*g*., AgaVba). We describe a simplified model of causal inference in multisensory speech perception (CIMS) that predicts the perception of arbitrary combinations of auditory and visual speech. We applied this model to behavioral data collected from 60 subjects perceiving both McGurk and non-McGurk incongruent speech stimuli. The CIMS model successfully predicted both the audiovisual integration observed for McGurk stimuli and the lack of integration observed for non-McGurk stimuli. An identical model without causal inference failed to accurately predict perception for either form of incongruent speech. The CIMS model uses causal inference to provide a computational framework for studying how the brain performs one of its most important tasks, integrating auditory and visual speech cues to allow us to communicate with others.

## Author Summary

During face-to-face conversations, we seamlessly integrate information from the talker’s voice with information from the talker’s face. This multisensory integration increases speech perception accuracy and can be critical for understanding speech in noisy environments with many people talking simultaneously. A major challenge for models of multisensory speech perception is thus deciding which voices and faces should be integrated. Our solution to this problem is based on the idea of causal inference—given a particular pair of auditory and visual syllables, the brain calculates the likelihood they are from a single *vs*. multiple talkers and uses this likelihood to determine the final speech percept. We compared our model with an alternative model that is identical, except that it always integrated the available cues. Using behavioral speech perception data from a large number of subjects, the model with causal inference better predicted how humans would (or would not) integrate audiovisual speech syllables. Our results suggest a fundamental role for a causal inference type calculation in multisensory speech perception.

## Introduction

Speech is the most important method of human communication and is fundamentally multisensory, with both auditory cues (the talker’s voice) and visual cues (the talker’s face) contributing to perception. Because auditory and visual speech cues can be corrupted by noise, integrating the cues allows subjects to more accurately perceive the speech content [1–3]. However, integrating auditory and visual speech cues can also lead subjects to *less* accurately perceive speech if the speech cues are incongruent. For instance, a unisensory auditory “ba” paired with a unisensory visual “ga” (AbaVga) leads to the perception of “da”, a speech stimulus that is not physically present. The illusion was first described experimentally by McGurk and MacDonald in 1976 [4] and is commonly known as the McGurk effect.

The McGurk effect has become a staple of classroom demonstrations and television documentaries because it is both simple and powerful—simply closing and opening one’s eyes completely changes the speech percept—and is also an important tool for research, with over 3,000 citations to the original paper in the last ten years. The McGurk effect is surprising because the incongruent speech tokens are easy to identify as physically incompatible: it is impossible for an open-mouth velar as seen in visual “ga” to produce a closed-mouth bilabial sound as heard in auditory “ba”. The effect raises fundamental questions about the computations underlying multisensory speech perception: Why would the brain integrate two incompatible speech components to produce an illusory percept? If the illusion happens at all, why does it not happen more often?

We propose a comprehensive computational model of multisensory speech perception that can explain these properties of the McGurk effect, building on previous models [2, 5–9]. Our model is based on the principle of *causal inference* [10–12]. Rather than integrating all available cues, observers should only integrate cues resulting from the same physical cause. In speech perception, humans often encounter environments with multiple faces and multiple voices and must decide whether to integrate information from a given face-voice pairing. More precisely, because observers can never be certain that a given face pairs with a given voice, they must infer the likelihood of each causal scenario (a single talker *vs*. separate talkers) and then combine the representations from each scenario, weighted by their likelihoods. For simple syllable perception, individuals often perceive the auditory component of speech when the face and voice are separate talkers [13]. The final result of causal inference is then the average of the integrated multisensory representation (the representation assuming a single talker) and the auditory representation (the representation assuming separate talkers), weighted by the likelihood that the face and voice arise from a single talker *vs*. separate talkers.

To test whether causal inference can account for the perception of incongruent multisensory speech, we created two similar models, one that *did* perform causal inference on multisensory speech (CIMS) and a model identical in every way, except that it *did not* perform causal inference (non-CIMS). We obtained predictions from the CIMS and non-CIMS models for a variety of audiovisual syllables and compared the model predictions with the percepts reported by human subjects presented with the same syllables.

## Methods

**Fig 1:**
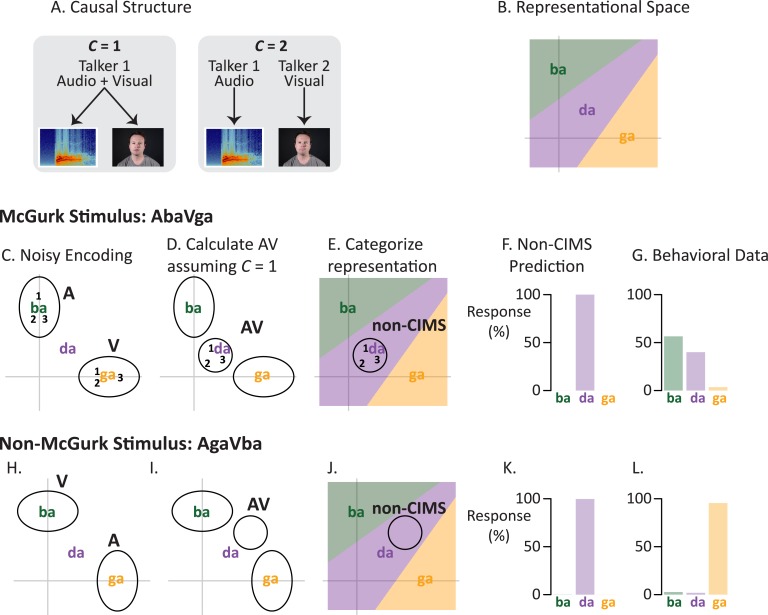
Modeling of multisensory speech perception without causal inference. (A) There are two possible causal structures for a given audiovisual speech stimulus. If there is a common cause (*C* = 1), a single talker generates the auditory and visual speech. Alternatively, if there is not a common cause (*C* = 2), two separate talkers generate the auditory and visual speech. (B) We generate multisensory representations in a two-dimensional representational space. The prototypes of the syllables “ba,” “da,” and “ga” (location of text labels) are mapped into the representational space with locations determined by pairwise confusability. The x-axis represents auditory features; the y-axis represents visual features. (C) Encoding the auditory “ba” + visual “ga” (AbaVga) McGurk stimulus. The unisensory components of the stimulus are encoded with noise that is independent across modalities. On three trials in which an identical AbaVga stimulus is presented (represented as 1, 2, 3) the encoded representations of the auditory and visual components differ because of sensory noise, although they are centered on the prototype (gray ellipses show 95% probability region across all presentations). Shapes of ellipses reflect reliability of each modality: for auditory “ba” (ellipse labeled A), the ellipse has its short axis along the auditory x-axis; visual “ga” (ellipse labeled V) has its short axis along the visual y-axis. (D) On each trial, the unisensory representations are integrated using Bayes’ rule to produce an integrated representation that is located between the unisensory components in representational space. Numbers show the actual location of the integrated unisensory representations from ***C***. Because of reliability weighting, the integrated representations are closer to “ga” along the visual y-axis, but closer to “ba” along the auditory x-axis (ellipse shows 95% probability region across all presentations). (E) Without causal inference (non-CIMS), the AV representation is the final representation. On most trials, the representation lies in the “da” region of representational space (numbers and 95% probability ellipse from **D**). (F) A linear decision rule is applied, resulting in a model prediction of exclusively “da” percepts across trials. (G) Behavioral data from 60 subjects reporting their percept of auditory “ba” + visual “ga”. Across trials, subjects reported the “ba” percept for 57% of trials and “da” for 40% of trials. (H) Encoding the auditory “ga” + visual “ba” (AgaVba) incongruent non-McGurk stimulus. The unisensory components are encoded with modality-specific noise; the auditory “ga” ellipse has its short axis along the auditory axis, the visual “ba” ellipse has its short axis along the visual axis. (I) Across many trials, the integrated representation (AV) is closer to “ga” along the auditory x-axis, but closer to “ba” along the visual *y*-axis. (J) Over many trials, the integrated representation is found most often in the “da” region of perceptual space. (K) Across trials, the non-CIMS model predicts “da” for the non-McGurk stimulus. (L) Behavioral data from 60 subjects reporting their perception of AgaVba. Subjects reported “ga” on 96% of trials.

In everyday environments, we encounter audiovisual speech and must decide whether the auditory and visual components of the speech emanate from a single talker (*C* = 1) or two separate talkers (*C* = 2; Fig 1A). Most studies of multisensory integration assume that *C* = 1 and focus on the details of the inference used to produce a single multisensory representation that is then categorized as a particular percept [2, 5]. To carry out causal inference, we must perform the additional steps of calculating the *C* = 2 representation and then combining the *C* = 1 and *C* = 2 representations. This combined representation is then categorized as a particular percept. Critically, identical stimuli can result in different percepts with (CIMS) and without (non-CIMS) causal inference.

We begin by describing the common elements of the CIMS and non-CIMS models. We define a two-dimensional space as the minimum possible dimension for characterizing auditory and visual speech information (Fig 1B). The x-axis represents auditory information in the stimulus while the y-axis represents visual information in the stimulus. For simplicity, we model a space containing only 3 speech token categories, “ba,” “da,” and “ga”. Based on behavioral confusability studies and previous modeling work [8, 14], “da” was placed intermediate to “ba” and “ga” on both the auditory and visual axes, slightly closer to “ba” on the auditory x-axis, and slightly closer to “ga” on the visual y-axis. These syllable locations can be thought of as prototypes, with different talkers (or different utterances from the same talker) differing from the prototype. To model these category distributions as simply as possible, we defined two-dimensional variance-covariance matrices (identical across syllables) with zero covariance (information in auditory and visual axes is uncorrelated) and equal variances along each axis. The axes of this representational space do not correspond in a simple way to physical properties of the stimulus; instead, they correspond to some internal neural representational space in which auditory and visual syllables are mapped into a single representational space that allows for integration.

A staple of Bayesian models of perception is the concept of sensory noise. Not only do individual exemplar stimuli vary from their prototype, the perceived stimulus varies from its actual physical properties due to sensory noise. We model this as two-dimensional variance-covariance matrices representing Gaussian noise in each modality (*Σ*_A_ and *Σ*_V_, for auditory and visual encoding) with variances inversely proportional to the precision of the modality. We chose Gaussian noise both because of its use in previous computational models [2, 5] and because Gaussian noise arises naturally in situations where a signal is corrupted by multiple independent sources. Modalities are encoded separately, but through extensive experience with audiovisual speech, encoding a unisensory speech stimulus provides some information about the other modality. For instance, hearing an unisensory auditory “ba” informs the observer that the mouth of the talker must have been in an initially lips-closed position. For such a unisensory cue, the information provided about the other sensory modality has higher variance. In our model, we assume that for auditory cues, the standard deviation along the visual axis is 1.5 times larger than the standard deviation along the auditory axis. For visual cues, the standard deviation along the auditory axis is 1.5 times larger than the standard deviation along the visual axis. This setup produces unisensory noise matrices that are rotations of one another (ellipses labelled **A** and **V** in Fig 1C).

For each presentation of a given audiovisual stimulus, the model encodes each modality separately. For a single trial of a stimulus with auditory component *S*_A_ and visual component *S*_V_, the model generates two vectors: the auditory representation $X_{A}∼N(S_{A},\Sigma_{A})$ and the visual representation $X_{V}∼N(S_{V},\Sigma_{V})$, where N(μ,Σ) is a normal distribution with mean *μ* and variance *Σ*. Across many trials, the values of *X*_A_ and *X*_V_ will cluster around the exemplar locations *S*_A_ and *S*_V_ with variance equal to the modality-specific encoding variances (Fig 1C, where *S*_A_ and *S*_V_ are the set to the prototypical representations for “ba” and “ga”, respectively).

To form the *C =* 1 representation, the model assumes Bayesian inference (integration of auditory and visual speech cues according to their reliabilities). We use the two-dimensional analog of the common Bayesian cue-integration rules as described by [2]. On each trial, we calculate the integrated representation as $X_{AV}=\Sigma_{AV}(\Sigma_{A}^{-1}X_{A}+\Sigma_{V}^{-1}X_{V})$, where $\Sigma_{AV}={\left(\Sigma_{A}^{-1}+\Sigma_{V}^{-1}\right)}^{-1}$. Across many trials, the distribution of the *C* = 1 representations will be the weighted average of the locations for *S*_A_ and *S*_V_ with the weighting controlled by the relative precision of the encoding matrices (Fig 1D).

Without causal inference, the integrated representation, *X*_AV_, is the final representation. Although the representational space is continuous, speech perception is categorical. Therefore, to produce a categorical percept, we determine the syllable that is most likely to have generated the integrated representation: $P(X_{A},X_{V}|S_{i})=N_{2}(X_{A,}X_{V};\mu_{S_{i},C=1},\Sigma_{S_{i},C=1})$, where $N_{2}$ is the two-dimensional Gaussian density function, $\mu_{S_{i},C=1}$ is the two-dimensional location of a particular syllable category and $\Sigma_{S_{i},C=1}=\Sigma_{i}+\Sigma_{AV}$ is the sum of the category’s variance-covariance matrix and the variance of *X*_AV_. For simplicity, we have assumed that all syllables have equal prior probability, all locations within the representational space have equal prior probability, and that the category variance-covariance matrices are equal. The resulting linear decision boundaries are shown in Fig 1E.

Across many trials, we can calculate the model responses for a given audiovisual stimulus. The frequency of each percept across simulated trials is tallied (Fig 1F) and the percentage of each percept is compared with behavioral data from human subjects (Fig 1G). All model simulations were done in R [15], multivariate probabilities were calculated using the *mvtnorm* package [16, 17]. Model predictions were generated based on 10,000 samples from each audiovisual syllable tested.

**Fig 2:**
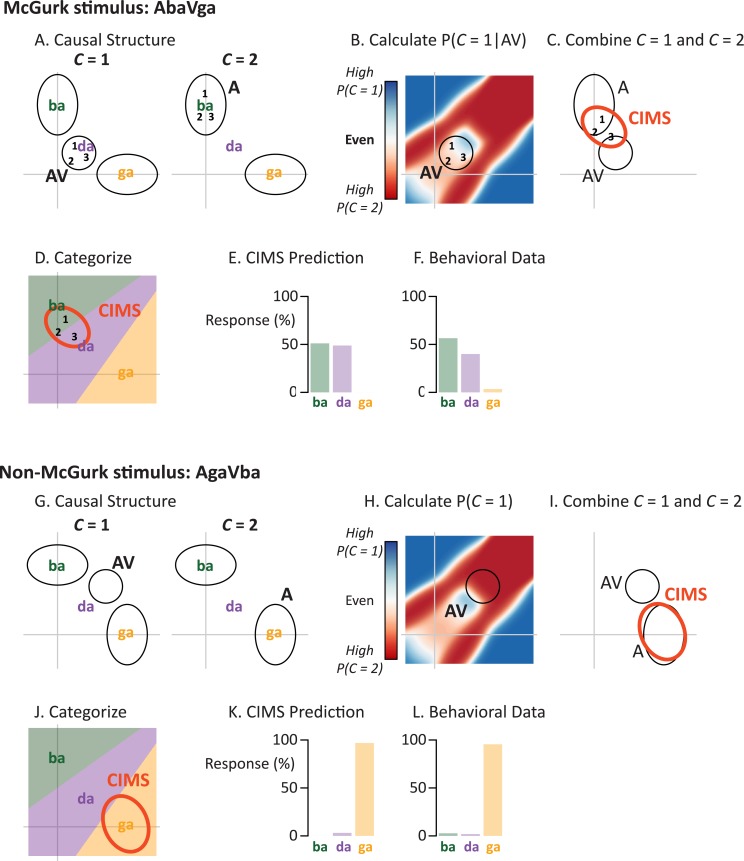
Modeling of multisensory speech perception with causal inference. (A) For the McGurk stimulus AbaVga, two causal structures are considered. If auditory and visual speech emanate from a single talker (*C* = 1), the integrated representation is calculated as in Fig 1***D***. If auditory and visual speech emanate from two talkers (*C* = 2) the representation is assumed to be the auditory component of the stimulus (Fig 1***C***). (B) The likelihood of each causal structure is calculated using knowledge about the natural statistics of audiovisual speech (red/blue color scale). Numbers (showing representation of an identical McGurk stimulus on three trials) are centered over the white region, indicating similar probability of *C* = 1 vs. *C* = 2 (ellipse shows 95% probability region across trials). (C) On each trial, a weighted average of the *C* = 1 (AV) and *C* = 2 (A) representations is calculated, using the probabilities from **B**. This shifts the representation so that it is intermediate between the AV and A representations (compare location of numbers with **B**). Red ellipse labelled CIMS shows 95% probability region across all trials. (D) A linear decision rule is applied to categorize the representation (same number and red ellipse as **C**). The CIMS representation is found most often in the “da” region of perceptual space, but sometimes lies in the “ba” region. (E) The model predicts “ba” for 51% of trials and “da” for 49% of trials. (F) Across trials, subjects reported the “ba” percept for 57% of trials and “da” for 40% of trials. (G) Causal inference for the non-McGurk auditory “ga” + visual “ba” (AgaVba) stimulus. The *C* = 1 representation (AV ellipse from Fig 1**I**) and the C = 2 representation (A ellipse from Fig 1**H**) are calculated. (H) The AV representations have a higher likelihood of originating from *C* = 2 (AV ellipse centered over red region). (I) Across many trials, the CIMS representations (red ellipse labelled CIMS is the 95% probability region) are shifted toward the *C* = 2 percept (A) away from the *C* = 1 representation (AV). (J) Over many trials, the CIMS representation is found most often in the “ga” region of perceptual space (same red ellipse as **I**). (K) The CIMS model primarily predicts “ga” (97%) for the non-McGurk AgaVba stimulus. (L) Behavioral data from 60 subjects reporting their perception of auditory “ga” + visual “ba.” Across trials, subjects reported “ga” for 96% of trials.

In the CIMS model, rather than assuming that *C* = 1, we take both the *C* = 1 and *C* = 2 representations into consideration, weighting them by their likelihood. For *C* = 1, the representation is the same as for the non-CIMS model (Fig 2A). For *C* = 2, the representation is simply the encoded representation of the auditory portion of the stimulus; this is reasonable because most incongruent pairings of auditory and visual speech result in perception of the auditory syllable [13]. Next, we calculate the log posterior ratio of *C* = 1 to *C = 2*:

$$d=\log\frac{P(C=1|X_{A},X_{V})}{P(C=2|X_{A},X_{V})}=\log\frac{P(X_{A},X_{V}|C=1)}{P(X_{A},X_{V}|C=2)}+\log\frac{P(C=1)}{P(C=2)}$$

The prior probability of a common cause, P(*C* = 1), is set to 0.50 (giving no prior bias toward one or two causes), resulting in P(*C* = 2) = 0.50. We calculate P(*X*_A_,*X*_*V*_|*C* = 1) by looking at each syllable, *S*_*i*_, individually. These probabilities are then combined, weighted by their respective prior probabilities (assumed to be equal) to determine the overall conditional probability:

$$P(X_{A},X_{V}|C=1)=\sum_{i}P(X_{A},X_{V}|S_{i})\pi_{S}(S_{i})$$

We calculate P(*X*_A_,*X*_*V*_|*C* = 2) using a similar process for all possible incongruent syllable combinations. Their locations in representational space are determined as

$$\mu_{S_{i},C=2}=\Sigma_{S_{i},C=2}(\Sigma_{A^{\prime }}^{-1}X_{A}+\Sigma_{V^{\prime }}^{-1}X_{V}),\;with\;\Sigma_{S_{i},C=2}={\left(\Sigma_{A^{\prime }}^{-1}+\Sigma_{V^{\prime }}^{-1}\right)}^{-1}$$

*X*_*A*_ and *X*_*V*_ are the locations for the unisensory components. The matrices *Σ*_A′_ and *Σ*_V′_ are the original sensory noise matrices plus the variance of the syllable category that generated the exemplar. The probabilities are then calculated using the two-dimensional Gaussian density: $N_{2}(X_{A,}X_{V};\mu_{S_{i},C=2},\Sigma_{S_{i},C=2})$ and weighted by their prior probabilities (assumed to be equal).

After the decision variable *d* is computed, we convert it into the probability of each causal structure (shown for all positions in the representational space in Fig 2B):

$$P(C=1|X_{A},X_{V})=\frac{1}{1+e^{-d}};\;P(C=2|X_{A},X_{V})=\frac{1}{1+e^{d}}$$

The next step is to combine the *C* = 1 and *C* = 2 representations, weighted by their likelihood (Fig 2C):

$$X_{CIMS}=P(C=1|X_{A},X_{V})X_{AV}+P(C=2|X_{A},X_{V})X_{A}$$

This combination is done on a trial-by-trial basis, producing a non-linear combination of the original noisily-encoded exemplars (*X*_A_ and *X*_V_). Across many trials the distribution of encoded exemplars is not multivariate Gaussian (an ellipse), but fitting a two-dimensional Gaussian to the exemplars provides a useful visual approximation for their distribution, depicting how the causal inference representations are intermediate between the *C* = 1 (AV) and *C* = 2 (A) representations (Fig 2C). For each trial, this representation is categorized into a percept using the same linear decision rule as for the model without causal inference (Fig 2D). The CIMS model responses across trials are computed (Fig 2E) and compared with human behavioral data (Fig 2F).

### Behavioral data

To test the predictions of each model, we collected syllable recognition data from 60 participants (18 female; mean age = 33) for 9 stimuli: all possible combinations of auditory and visual “ba”, “da” and “ga” from a single talker. Participants were recruited using Amazon Mechanical Turk, using an identical design and procedure as a previous study of McGurk perception [18]. Each stimulus was repeated 10 times in a randomized order. Participants reported their percept of each audiovisual syllable by selecting from among: “ba”, “da/tha”, and “ga”. Performance on congruent syllables was at or near ceiling for all subjects (mean accuracy = 97%; range 87% to 100%).

## Results

We constructed two similar computational models of audiovisual speech perception that differed only in whether they did (CIMS) or did not (non-CIMS) incorporate causal inference. While the non-CIMS model assumes a common cause, the CIMS model estimates the likelihood of common *vs*. separate causes for each trial (Fig 1A). We calculated the predictions of the models for two types of incongruent speech, McGurk syllables and non-McGurk syllables, and compared the predictions with behavioral data collected from human subjects.

First, we examine the non-CIMS model. A McGurk stimulus consisting of an auditory “ba” and visual “ga” is represented in the model by a two-dimensional representational space in which the x-axis represents visual information and the y-axis represents auditory information (Fig 1B). The model assumes that the auditory “ba” and visual “ga” emanate from a single talker (*C* = 1) and integrates the unisensory cues according to Bayesian principles, with each cue weighted by its reliability. This results in an integrated audiovisual representation that is closer to “ba” along the auditory axis and closer to “ga” along the visual axis (Fig 1D). After applying a linear categorization rule, the multisensory percept lies in the “da” region of perceptual space (Fig 1E). Across multiple trials, identical physical stimuli are encoded differently because of sensory noise, resulting in a distribution of representations. Because speech is categorical, this variability can still lead to identical percepts; in this case, the model predicts a percept of “da” on >99% of trials and “ba” or “ga” on <1% of trials (Fig 1F). Comparing this result to behavioral data collected from 60 human subjects (Fig 1G) shows a poor correspondence. Subjects reported the “da” percept on only 40% of trials, and the “ba” percept on 57% of trials.

The non-CIMS model’s correspondence with human perception was even worse for a non-McGurk syllable consisting of an auditory “ga” and visual “ba” (Fig 1K and 1L). This stimulus is the reverse of the McGurk syllable (auditory “ba” and visual “ga”) so that when the two unisensory cues were integrated according to Bayesian principles, the audiovisual representation was placed in a different region of perceptual space than the McGurk syllable (Fig 1I). However, applying the linear categorization rule meant that the model still classified the percept as “da” (Fig 1J) on >99% of trials (Fig 1K). This prediction was inaccurate, as human subjects reported “da” on 2% of trials, instead reporting “ga” on 96% of trials (Fig 1L).

Next, we examined the predictions of the CIMS model. The noisy encoding, Bayesian integration, and categorization modules were identical to the non-CIMS model, but an additional step of causal inference was performed. For any given speech stimulus, the true causal structure is unknown, meaning that the optimal strategy is to estimate the *C* = 1 and *C* = 2 representations (Fig 2A) and combine them, weighted by their probabilities.

For a McGurk stimulus, the audiovisual representation lies in a region in which *C* = 1 and *C* = 2 are equally likely (Fig 2B). This results in a CIMS representation located between the *C* = 1 (audiovisual, “da”) and *C* = 2 representations (auditory, “ba”) (Fig 2C). Applying the categorization rule to the representations generated across many trials (Fig 2D), the CIMS model predicts a mixture of “ba” percepts (51%) and “da” percepts (49%; Fig 2E). This prediction is a good match to the behavioral data (57% “ba”, 40% “ba”; Fig 2F).

For a non-McGurk stimulus (Fig 2G), the audiovisual representation is much more likely to be obtained from the *C* = 2 than the *C* = 1 distribution (Fig 2H). Therefore, the output of the causal inference step is much more strongly weighted towards the *C* = 2 representation (auditory, “ga”) than the *C* = 1 representation (audiovisual, “da”) (Fig 2I). Applying the categorization rule to the causal inference representations generated across many trials (Fig 2J), the model predicts predominantly “ga” percepts (97%; Fig 2K) as was observed in the behavioral data (96% “ga”; Fig 2L).

### Testing with other syllable combinations

**Fig 3:**
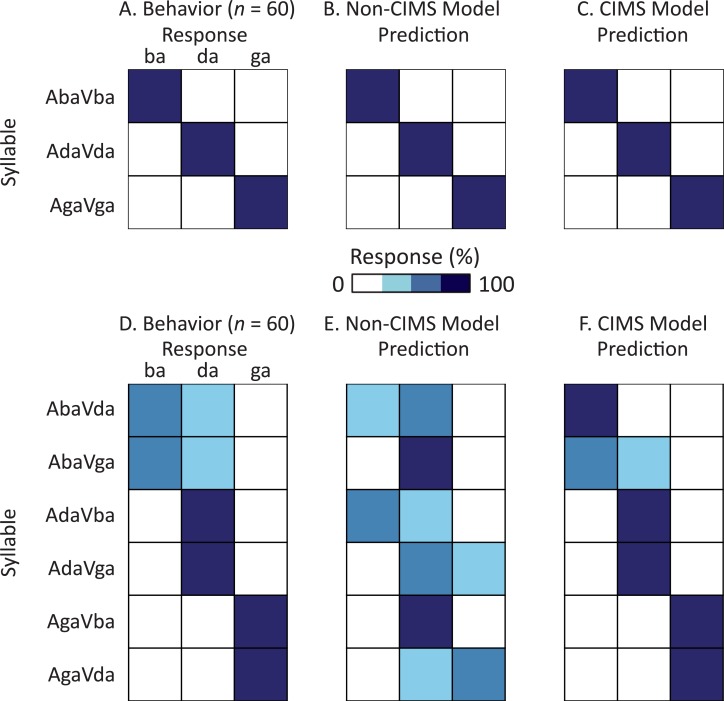
Generalizability of models tested with other audiovisual syllables. (A) Behavior for congruent syllables. Each row represents a different congruent audiovisual syllable (AbaVba, AdaVda, AgaVga). Subjects made a three-alternative forced choice (ba, ga, da). The colors within each row show how often subjects reported each choice when presented with each syllable (*e*.*g*. for AbaVba, they always reported “ba”). (B) Non-CIMS model predictions for congruent syllables. Rows show syllables, colors across columns within each row show how often model predicted that percept (darker colors indicate higher percentages). (C) CIMS model predictions for congruent syllables. (D) Behavior for incongruent syllables. Each row represents a different incongruent audiovisual syllable. Subjects made a three-alternative forced choice (ba, ga, da). The colors within each row show how often subjects reported each choice when presented with each syllable (*e*.*g*. for AbaVda, they more often reported “ba”, less often reported “da”, never reported “ga”). (E) Non-CIMS model predictions for incongruent syllables. Rows show syllables, colors across columns within each row show how often model predicted that percept (darker colors indicate higher percentages). (F) CIMS model predictions for incongruent syllables.

To assess generalizability, we also tested both CIMS and non-CIMS models with other audiovisual syllables (Fig 3). For congruent syllables (AbaVba, AdaVda, AgaVga) human subjects show little variability, always reporting the syllable that is present in both modalities (Fig 3A). Both the non-CIMS (Fig 3B) and CIMS (Fig 3C) models predict this behavior with excellent fidelity (correlation between behavior and model prediction *r* ≈ 1 for both). However, differences between the predictions of the two models appear when they are tested with incongruent audiovisual syllables, with only the CIMS model able to accurately predict human perception. Across six different incongruent syllables, the CIMS model showed a significantly stronger correlation with human perception (correlation between behavioral and model: CIMS *r* = 0.95 *vs*. non-CIMS *r* = 0.21, *z* = 4.3, *p* = 10^−5^ from Fisher r-to-z transformation.)

## Discussion

These results are important because speech is the most important form of human communication and is fundamentally multisensory, making use of both visual information from the talker’s face and the auditory information from the talker’s voice. In everyday situations we are frequently confronted with multiple talkers emitting auditory and visual speech cues, and the brain must decide whether or not to integrate a particular combination of voice and face. The best known laboratory example of this situation is the McGurk effect, in which an incongruent auditory “ba” and visual “ga” are fused to result in the percept “da”. This simple yet powerful illusion has been used in thousands of studies, ranging from developmental to clinical, to intercultural. However, there has been no clear theoretical understanding of why the McGurk effect occurs for some incongruent syllables (e.g. AbaVga) but not others (e.g. AgaVba). Some process must be operating that distinguishes between incongruent audiovisual speech that *should* and *should not* be integrated. A quantitative framework for this process is provided by causal inference.

We constructed two similar computational models of audiovisual speech perception. The CIMS and non-CIMS models, although identical in every respect except for the inclusion of causal inference, generated very different predictions about the perception of incongruent syllables. When tested with McGurk and inverse-McGurk incongruent syllables, the non-CIMS model predicted exclusively “da” responses. This was an inaccurate description of the perceptual reports of human subjects, who reported distinct percepts for the two types (mixture of “da” and “ba” for McGurk syllables *vs*. exclusively “ga” for inverse-McGurk syllables). In contrast, the CIMS model successfully reproduced this pattern of perceptual reports. In a test of generalizability, the CIMS model also was able to better predict perception than the non-CIMS model for six other incongruent audiovisual syllables.

The comparison between CIMS and non-CIMS models was fair because (except for causal inference) they were identical across all model steps, including the layout of the representational space, the amount of encoding noise, the rule used to integrate auditory and visual cues, and the rule used to categorize the final representations. We did not explicitly optimize the model parameters to the behavioral data for either model, simply choosing the values heuristically or setting them at plausible defaults (e.g., flat priors). Thus, any difference in performance between the CIMS and non-CIMS models is attributable to causal inference, not to over-fitting (the CIMS model has only one extra free parameter). There is no way for the non-CIMS model to accurately predict behavior for both McGurk and inverse McGurk stimuli without imposing arbitrary rules about integration, which is, after all, the rationale for considering causal inference in the first place.

Similarly, the precise stimuli and subjects used to generate the behavioral data was also not a key factor in the better performance of the CIMS model. Although there is variability in the frequency with which different subjects report the illusory McGurk percept and the efficacy of different stimuli in evoking it [18, 19], we are not aware of any reports of the inverse McGurk stimuli evoking an illusory percept, as predicted by the non-CIMS model. Without a way to predict integration for some combination and segregation for others, the non-CIMS model simply cannot replicate the observed pattern of human syllable recognition.

### Model predictions

A key reason for creating models of cognitive processes is to generate testable predictions. The CIMS model successfully predicted perception for arbitrary combinations of three auditory and visual syllables (“ba”, “da”, and “ga”). By extending the representational space to consider other factors (e.g., voice onset time) the CIMS model could predict perception for any combination of auditory and visual syllables.

The CIMS model is also extensible to other cues that provides information about the causal structure of the stimulus. For the incongruent speech considered in the present paper, the main cue for causal inference is the content of the auditory and visual speech. However, there are other useful cues that can be used to estimate whether auditory and visual speech emanate from the same talker, especially the temporal disparity between auditory and visual speech [20–23]. As the delay between auditory and visual speech is increased, observers are more likely to judge that they emanate from different talkers [24]. Causal inference predicts that observers should be less likely to integrate incongruent auditory and visual speech at high temporal disparity than at low disparity, and this is indeed the case: the McGurk percept of “da” for AbaVga stimuli is reported less frequently as temporal disparity increases [25–27]. This phenomenon could be incorporated into the CIMS model by adding an additional dimension to the common-cause computation, allowing for independent estimates of P(*C* = 1) for any given speech content disparity or temporal disparity. A similar extension would be possible for the different syllable exemplars of the same syllable combination generated from different talkers. One talker’s “ga” might provide more or less visual speech information than another talker’s, driving P(*C* = 1) and the frequency of the McGurk effect higher or lower. Some evidence for this idea is supported by data showing that detection of audiovisual incongruence is correlated with McGurk perception [28].

A key direction for future research will also be a better understanding of the neural mechanisms underlying causal inference in speech perception. The CIMS model predicts that the ultimate percept of multisensory speech results from a combination of the *C* = 1 (AV) and *C* = 2 (A) representations. This requires that the brain must contain distinct neural signatures of both *C* = 1 and *C* = 2 representations. In an audiovisual localization task, there is fMRI evidence for *C* = 2 representations in early sensory cortex and a *C* = 1 representation in the intraparietal sulcus [29]. For multisensory speech, the *C* = 1 (AV) representation is most likely represented in the superior temporal sulcus (STS) because interrupting activity in the STS interferes with perception of the McGurk effect [30] and the amplitude of STS activity measured with fMRI predicts McGurk perception in adults [31] and children [32].

### Relationship with other models

McGurk and MacDonald offered a descriptive word model of the illusion, stating that for AbaVga “there is visual information for [ga] and [da] and auditory information with features common to [da] and [ba]. By responding to the common information in both modalities, a subject would arrive at the unifying percept [da].” [4]. The CIMS model differs from this word model in several fundamental respects. Unlike the word model, the CIMS model is both quantitative, allowing for precise numerical predictions about behavior, and probabilistic, allowing percepts to vary from trial-to-trial of the identical stimuli as well as across different stimuli and observers. This allows it to more accurately describe actual human perception. For instance, the word model predicts that every AbaVga stimulus will be perceived as “da” while behavioral data show a wide range in efficacy for different AbaVga stimuli [18, 19].

The fuzzy logical model of perception (FLMP) as developed by Massaro [7, 33] was an important advance because it was one of the first probabilistic models, allowing successive presentations of identical stimuli to produce different percepts. However, because the FLMP does not explicitly model the processes underlying perception, it has no way to separate stimulus variability (the location in representational space for CIMS) and sensory noise (the ellipse describing the distribution of encoded representations for CIMS).

Another important model of the McGurk effect uses predictive coding [8]. While the representational space in this model is somewhat similar to that of the CIMS model, the predictive coding model is a multi-level network model that allows for dynamic prediction of perception as evidence from different sensory modalities arrives asynchronously. However, because it does not incorporate sensory noise it cannot account for trial-to-trial differences in perception of identical stimuli.

The noisy encoding of disparity (NED) model used three parameters to account for trial-to-trial differences in perception [6]. However, the NED model predicts only one of two pre-specified percepts resulting from either the presence or absence of integration. The CIMS model is a significant advance over the NED model because it allows for a continuous variation along the axis from complete integration to complete segregation, and can thus produce the percept of any stimulus within the representational space.

### Other variables impacting causal inference

The role of causal inference in multisensory speech has been previously considered within the context of a synchrony judgment task [24]. Although this model is a Bayesian causal inference model, it has only superficial similarity to the current model. The earlier model focused on how an observer could use causal inference to decide if two signals produced at distinct points in time were generated from the same talker. The input to the model was thus a fixed asynchrony and the output a binary judgment of synchronous *vs*. asynchronous. In contrast, the current model does not consider the temporal relationship between the auditory and visual syllables, but rather is concerned only with their content and outputs a perceived syllable. In principle a more complicated that considers content and temporal disparity jointly.

Previous research has shown that not all kinds of disparity are used to determine how much to integrate auditory and visual speech streams. Previous researchers have created McGurk stimuli in which the auditory sounds and visual faces were from talkers with different genders [34]. Even though participants were able to identify this discrepancy, the McGurk effect was not diminished compared to stimuli with talkers from the same gender. This study highlights the important distinction between the perceived speech, and the judgment of a common cause *per se*.

There are at least two ways to square the idea that causal inference is important for integration but ignores information that would seemingly inform the calculation. First, even when the probability of a common cause is low, some weight is still given to the multisensory representation and thus a “da” percept is still possible. For instance, on a given trial where the probability of a common cause is 0.4, the optimal report from the participant is that there are 2 talkers. However, the final representation will still be given a substantial weight and thus the percept could be driven to a fusion response, rather than an auditory report. Second, there may be other, stronger indicators of a common cause that can override a noted discrepancy in a particular feature. For instance, the temporal simultaneity and the spatial compatibility of the auditory and visual tokens may together make the *C* = 1 scenario plausible, despite an apparent mismatch between the perceived gender of the auditory and visual speech. Well-studied phenomenon like the ventriloquist illusion [10, 35, 36] provide strong evidence that temporal cues elicit strong control of integration despite spatial disparity that would otherwise indicate separate talkers. These options are not mutually exclusive. A study of how temporal synchrony affect McGurk perception suggest both explanations may be at play. In one study, researchers measured perception of synchrony and perceived speech using both congruent and McGurk stimuli [27]. Reported synchrony was lower for McGurk stimuli than for congruent stimuli, consistent with the use of a combined disparity measure. In a separate study, the McGurk effect was perceived at asynchronies these same subjects judge to be asynchronous, and ostensibly not uttered by the same talker [25]. Taken together, these studies show that the general framework of causal inference can be fruitfully explored, and that a major issue for future studies is to determine the relative weighting of stimulus features in estimating the likelihood of a common cause.

### Generalized causal inference

The CIMS model is a simplification of a full Bayes-optimal causal inference model. For instance, the CIMS calculation of the final percept ignores differences in the prior for different locations within the representational space and the CIMS estimates of the likelihood of each causal structure are calculated using only integrated location (AV) rather than the joint distribution of the individual cues (A and V).

While our results suggest that causal inference is a key step in audiovisual speech perception, there are many possible solutions to the general problem of causal inference [37]. The CIMS model solves the problem by combining the *C* = 1 and *C* = 2 representations according to their probability (sometimes known as a “weighted average” or “model averaging” approach). A second option is to select the percept that is most likely on each single trial (“winner-take-all” or “model selection”). A third option is to distribute choices between *C* = 1 and *C* = 2 based on their probability (“probability matching”). The categorical nature of speech perception means that a very large stimulus set (much larger than that used in the present study) would be needed to determine which solution or solutions is used for audiovisual speech perception by human subjects. Here, we focused on model averaging because it was shown to be successful in previous studies of audiovisual perception in human subjects [10, 24].

## References

[1] Knill DC, Pouget A. The Bayesian brain: the role of uncertainty in neural coding and computation. Trends Neurosci. 2004;27(12):712–9. 10.1016/j.tins.2004.10.007 15541511

[2] Ma WJ, Zhou X, Ross LA, Foxe JJ, Parra LC. Lip-reading aids word recognition most in moderate noise: a Bayesian explanation using high-dimensional feature space. PLoS One. 2009;4(3):e4638 Epub 2009/03/05. PubMed Central PMCID: PMC2645675. 10.1371/journal.pone.0004638 19259259 PMC2645675

[3] Sumby WH, Pollack I. Visual contribution to speech intelligibility in noise. J Acoust Soc Am. 1954;26(2):212–5.

[4] McGurk H, MacDonald J. Hearing lips and seeing voices. Nature. 1976;264(5588):746–8. 1012311 10.1038/264746a0

[5] Bejjanki VR, Clayards M, Knill DC, Aslin RN. Cue integration in categorical tasks: insights from audio-visual speech perception. PLoS One. 2011;6(5):e19812 Epub 2011/06/04. PubMed Central PMCID: PMC3102664. 10.1371/journal.pone.0019812 21637344 PMC3102664

[6] Magnotti JF, Beauchamp MS. The noisy encoding of disparity model of the McGurk effect. Psychonomic Bulletin & Review. 2015;22(3):701–9. 25245268 10.3758/s13423-014-0722-2 PMC4370809

[7] Massaro DW. Perceiving talking faces: from speech perception to a behavioral principle Cambridge, Mass.: MIT Press; 1998 xii, 494 p. p.

[8] Olasagasti I, Bouton S, Giraud A-L. Prediction across sensory modalities: A neurocomputational model of the McGurk effect. Cortex. 2015;68:61–75. 10.1016/j.cortex.2015.04.008 26009260

[9] Schwartz JL. A reanalysis of McGurk data suggests that audiovisual fusion in speech perception is subject-dependent. J Acoust Soc Am. 2010;127(3):1584–94. 10.1121/1.3293001 20329858

[10] Kording KP, Beierholm U, Ma WJ, Quartz S, Tenenbaum JB, Shams L. Causal inference in multisensory perception. PLoS One. 2007;2(9):e943 Epub 2007/09/27. PubMed Central PMCID: PMC1978520. 10.1371/journal.pone.0000943 17895984 PMC1978520

[11] Shams L, Beierholm UR. Causal inference in perception. Trends Cogn Sci. 2010;14(9):425–32. Epub 2010/08/14. 10.1016/j.tics.2010.07.001 20705502

[12] Schutz M, Kubovy M. Causality and cross-modal integration. J Exp Psychol Hum Percept Perform. 2009;35(6):1791–810. Epub 2009/12/09. 10.1037/a0016455 19968437

[13] MacDonald J, McGurk H. Visual influences on speech perception processes. Percept Psychophys. 1978;24(3):253–7. 704285 10.3758/bf03206096

[14] Liberman AM, Delattre PC, Cooper FS, Gerstman LJ. The role of consonant-vowel transitions in the perception of the stop and nasal consonants. Psychological Monographs: General and Applied. 1954;68(8):1.

[15] R Core Team. R: A language and environment for statistical computing Vienna, Austria: R Foundation for Statistical Computing; 2015.

[16] Genz A, Bretz F. Computation of Multivariate Normal and t Probabilities Lecture Notes in Statistics. 195 Heidelberg: Springer-Verlag; 2009.

[17] Genz A, Bretz F, Miwa T, Mi X, Leisch F, Scheipl F, et al. mvtnorm: Multivariate Normal and t Distributions. R package version 1.0–5. 2016.

[18] Basu Mallick D, Magnotti JF, Beauchamp MS. Variability and stability in the McGurk effect: contributions of participants, stimuli, time, and response type. Psychonomic bulletin & review. 2015:1–9. 25802068 10.3758/s13423-015-0817-4 PMC4580505

[19] Jiang J, Bernstein LE. Psychophysics of the McGurk and other audiovisual speech integration effects. J Exp Psychol Hum Percept Perform. 2011;37(4):1193–209. PubMed Central PMCID: PMC3149717. 10.1037/a0023100 21574741 PMC3149717

[20] Chandrasekaran C, Trubanova A, Stillittano S, Caplier A, Ghazanfar AA. The natural statistics of audiovisual speech. PLoS Comput Biol. 2009;5(7):e1000436 Epub 2009/07/18. PubMed Central PMCID: PMC2700967. 10.1371/journal.pcbi.1000436 19609344 PMC2700967

[21] Conrey B, Pisoni DB. Auditory-visual speech perception and synchrony detection for speech and nonspeech signals. J Acoust Soc Am. 2006;119(6):4065–73. Epub 2006/07/15. PubMed Central PMCID: PMC3314884. 16838548 10.1121/1.2195091 PMC3314884

[22] Dixon NF, Spitz L. The detection of auditory visual desynchrony. Perception. 1980;9(6):719–21. 7220244 10.1068/p090719

[23] Vroomen J, Keetels M. Perception of intersensory synchrony: a tutorial review. Atten Percept Psychophys. 2010;72(4):871–84. Epub 2010/05/04. 10.3758/APP.72.4.871 20436185

[24] Magnotti JF, Ma WJ, Beauchamp MS. Causal inference of asynchronous audiovisual speech. Frontiers in Psychology. 2013;4:798 10.3389/fpsyg.2013.00798 24294207 PMC3826594

[25] Soto-Faraco S, Alsius A. Deconstructing the McGurk–MacDonald illusion. J Exp Psychol Hum Percept Perform. 2009;35(2):580 10.1037/a0013483 19331510

[26] Munhall KG, Gribble P, Sacco L, Ward M. Temporal constraints on the McGurk effect. Perception & psychophysics. 1996;58(3):351–62. Epub 1996/04/01. 8935896 10.3758/bf03206811

[27] van Wassenhove V, Grant KW, Poeppel D. Temporal window of integration in auditory-visual speech perception. Neuropsychologia. 2007;45(3):598–607. 10.1016/j.neuropsychologia.2006.01.001 16530232

[28] Strand J, Cooperman A, Rowe J, Simenstad A. Individual Differences in Susceptibility to the McGurk Effect: Links With Lipreading and Detecting Audiovisual Incongruity. Journal of Speech, Language, and Hearing Research. 2014;57(6):2322–31. 10.1044/2014_JSLHR-H-14-0059 25296272

[29] Rohe T, Noppeney U. Cortical hierarchies perform Bayesian causal inference in multisensory perception. PLoS Biol. 2015;13(2):e1002073 10.1371/journal.pbio.1002073 25710328 PMC4339735

[30] Beauchamp MS, Nath AR, Pasalar S. fMRI-Guided transcranial magnetic stimulation reveals that the superior temporal sulcus is a cortical locus of the McGurk effect. J Neurosci. 2010;30(7):2414–7. Epub 2010/02/19. PubMed Central PMCID: PMC2844713. 10.1523/JNEUROSCI.4865-09.2010 20164324 PMC2844713

[31] Nath AR, Beauchamp MS. A neural basis for interindividual differences in the McGurk effect, a multisensory speech illusion. NeuroImage. 2012;59(1):781–7. Epub 2011/07/27. PubMed Central PMCID: PMC3196040. 10.1016/j.neuroimage.2011.07.024 21787869 PMC3196040

[32] Nath AR, Fava EE, Beauchamp MS. Neural correlates of interindividual differences in children's audiovisual speech perception. The Journal of neuroscience: the official journal of the Society for Neuroscience. 2011;31(39):13963–71. Epub 2011/10/01. PubMed Central PMCID: PMC3203203. 21957257 10.1523/JNEUROSCI.2605-11.2011 PMC3203203

[33] Massaro DW. Testing between the TRACE model and the fuzzy logical model of speech perception. Cogn Psychol. 1989;21(3):398–421. Epub 1989/07/01. 2758786 10.1016/0010-0285(89)90014-5

[34] Green KP, Kuhl PK, Meltzoff AN, Stevens EB. Integrating speech information across talkers, gender, and sensory modality: female faces and male voices in the McGurk effect. Percept Psychophys. 1991;50(6):524–36. 1780200 10.3758/bf03207536

[35] Jack CE, Thurlow WR. Effects of degree of visual association and angle of displacement on the" ventriloquism" effect. Perceptual and motor skills. 1973. 10.1177/003151257303700360 4764534

[36] Warren DH, Welch RB, McCarthy TJ. The role of visual-auditory “compellingness” in the ventriloquism effect: Implications for transitivity among the spatial senses. Perception & Psychophysics. 1981;30(6):557–64. 7335452 10.3758/bf03202010

[37] Wozny DR, Beierholm UR, Shams L. Probability matching as a computational strategy used in perception. PLoS Comput Biol. 2010;6(8). Epub 2010/08/12. PubMed Central PMCID: PMC2916852. 10.1371/journal.pcbi.1000871 20700493 PMC2916852

